# Comment on “Disulfiram as an anti-inflammatory agent: mechanisms, nano-delivery strategies, and applications in non-oncologic diseases” by Q. Jiang, M. Jiang, Y. Lv, X. Zhang, S. Wang and J. Zhao, *RSC Adv.*, 2025, **15**, 36344

**DOI:** 10.1039/d6ra02136k

**Published:** 2026-07-09

**Authors:** Boris Cvek

**Affiliations:** a Palacky University Olomouc Czech Republic cvekb@seznam.cz

## Abstract

The referenced article reviews the purported anti-inflammatory properties of disulfiram, an anti-alcohol drug also known as Antabuse. However, the anti-inflammatory properties of disulfiram should be considered alongside a wealth of scientific research demonstrating its potential to treat various conditions, including cancers, Alzheimer’s disease, sepsis, tuberculosis, blindness and heart conditions. I argue that almost all these results are unreliable using basic chemical knowledge. Most importantly, disulfiram has never been shown to be an active molecule *in vivo*. The authors of the referenced article did not address this fact at all. Testing disulfiram *in vitro* yields misleading and dangerous pseudoscientific “results”, suggesting that it could be a potential treatment for an ever-growing list of diseases.

## Abstract 2

Will the real hero please step forward? Diethylthiomethyl carbamate, a metabolite of disulfiram (Antabuse) blocks ALDH while disulfiram itself has no proven biological activity.

## Graphical abstract

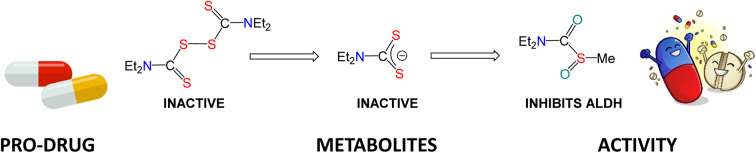

## Introduction^1^

Disulfiram was discovered by chance to block aldehyde dehydrogenase (ALDH) in the liver; the enzyme is responsible for converting acetaldehyde, a product of alcohol metabolism, into acetic acid. Consequently, alcoholics taking disulfiram (effervescent pills under brand name Antabuse) experience unpleasant symptoms when they drink alcohol, and this effect has been utilized in alcohol-aversion therapy for decades.

**Fig. 1:**
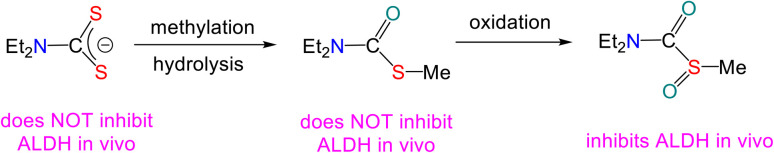
Non-active and active metabolites of disulfiram in ALDH inhibition.

However, it is important to note that disulfiram does not directly block enzymes in the body; multiple metabolizations are required. Thus, disulfiram is a highly reactive compound which is rapidly metabolized in the human body to diethyl-dithiocarbamate (first formula in Fig. 1). However, this is not an active ALDH blocker, rather it must be further metabolized to afford the true active entity (Fig. 1). Therefore, any reliable research on repurposing disulfiram must identify the metabolite responsible for the effects observed when testing disulfiram *in vivo* before any sensible mechanistic understanding can be addressed. In the case of disulfiram, due to its reactivity and rapid metabolism, it is certain that disulfiram itself is never the active component.

## *In vitro* panacea

In a recent issue of *RSC Advances*, Jiang *et al.*^2^ attempt to review publications related to the purported anti-inflammatory activity of disulfiram. While this review has some merit, in that the paper search has been done thoroughly, the lack of any insight and critical evaluation of the publications is a serious error. Thus, Jiang *et al.* simply accept the word of others that disulfiram is itself an active anti-inflammatory, rather than relating observed activity to metabolites.

Any review article on disulfiram as a therapeutic agent must critically discuss how this seemingly simple molecule can allegedly treat an ever-growing list of apparently unrelated diseases, including sepsis, cancer, Alzheimer’s disease, infections (such as tuberculosis, SARS-CoV-2, and Lyme disease), and obesity.

Disulfiram has never been identified as an active chemical entity *in vivo*. In order to even begin to elucidate a mechanism of action, a chemically defined entity that is demonstrably an active compound *in vivo* is a required starting point, since necessarily the elucidated mechanism pertains to it (see the first rule in “The Rules Often Neglected in Current Medicinal Chemistry”).^3^ For example, there can be no mechanism of action of disulfiram inhibiting ALDH *in vivo* because disulfiram does not inhibit ALDH *in vivo*. Indisputably, a non-existent action cannot have a mechanism.

Indeed, testing disulfiram *in vitro* could alter hundreds of proteins due to its reactivity, creating the illusion of a panacea with a plethora of unrelated and even contradictory mechanisms of action. Disulfiram can effectively treat almost any *in vitro* disease model, with higher concentrations (*i.e.*, 10 to 100 times greater than the physiologically meaningful)^1^ and longer treatment times (*i.e.*, tens of hours) simply serving to increase the probability of observing an effect. This is even before its metal complexation is taken into account, especially with the presence of copper in the medium.^4^

Thus, simple logic dictates that if it has never been demonstrated that disulfiram is an active drug molecule then there is no reason to use this compound for experiments *in vitro*. All this work – hundreds of articles published in the scientific literature – constructs a labyrinth of mechanisms of action that have no physiological relevance. These mechanisms are simply an artificial byproduct of laboratory models designed to produce “scientific results”. While Jiang *et al.*^2^ might argue that they are only the messenger, to write a review that fails to critically evaluate the literature is to pour petrol onto flames that are already burning strongly. A worrying consequence of this is that others follow blindly leading to clear errors being perpetuated to (seemingly) become truth.

## Conclusions

Most in science understand that bibliometric citations (*e.g.*, those on Web of Science) do not indicate whether the cited article has been fully read or understood, and too often, the answer to both is no (this not only highlights the absurdity of citation indexes but also potentially brings about long-term damage to scientific pursuit). When faced with such practice we have two options. The first is to get angry and frustrated but ultimately accept that such things happen, the second is to seek to address the obvious error (once again: non-existent action cannot have a mechanism) in print in the hope that an important message will finally be heard and accepted.

Taking a broader view, any serious review article should analyze the situation in the field and provide an overview of the latest developments, showing how various pieces of research fit together, contradict each other, and allow conclusions to be drawn or highlight areas that need further investigation. Once again, the first question that needs answering when it comes to disulfiram is how it can treat so many distinct diseases *via* a variety of unrelated mechanisms of action. Is all this research (*i.e.* testing disulfiram *in vitro*) merely a by-product of laboratory models? Yes, it is.

The most important context of the disulfiram saga is that it is inexpensive drug, readily available and its biological properties have been widely studied. Based on unreliable articles, it could immediately be used to “help” patients with sepsis, Alzheimer’s disease, tuberculosis, and many other conditions. This is a very dangerous situation. During the pandemic, the Ministry of Health in the Czech Republic ordered the removal of a YouTube video claiming that disulfiram was an effective treatment for COVID-19. In the case of metastatic cancer, the situation is different,^1^ as two randomized, double-blind, placebo-controlled phase II clinical trials have shown promising results and the active compound, and its mechanism of action, have been identified.

## Conflicts of interest

There are no conflicts to declare.

## Data Availability

No primary research results, software or code have been included and no new data were generated or analysed as part of this comment.
